# RORγt^+^ APCs require a distinct cis-regulatory element to instruct tolerance to dietary antigens

**DOI:** 10.21203/rs.3.rs-4865841/v1

**Published:** 2025-08-01

**Authors:** Xiaohuan Guo, Jie Zhao, Jiacheng Hao, Jincheng Chen, Mengze Lyu, Haoyu Liu, Na Li, Panwei Song, Wenyan Wang, Coco Chu, Gregory Sonnenberg

**Affiliations:** Institute for Immunology, School of Medicine, Tsinghua University; Tsinghua University; Tsinghua University; Institute for Immunology, School of Medicine, Tsinghua University; Weill Cornell Medical College; Tsinghua University; Institute for Immunology, School of Medicine, Tsinghua University; Tsinghua University; Tsinghua University; Tsinghua University; Cornell University

## Abstract

Oral tolerance represents a hallmark of intestinal mucosal immunity to prevent inflammatory responses to harmless natural antigens, such as dietary components or commensal organisms. However, the underlying mechanisms governing oral tolerance remain incompletely understood. Recent studies have shown that RORγt^+^ antigen-presenting cells (APCs) contribute to intestinal homeostasis through inducing microbiota-specific Tregs. Whether RORγt^+^ APCs can regulate dietary antigen-specific Tregs and thereby mediating oral tolerance remains unclear. Here, by comparing ATAC-seq data within *Rorc* gene loci between RORγt^+^ cell types, we identified a distinct cis-regulatory element, OCR369, which specifically regulates RORγt expression in ILC3s and other RORγt^+^ APCs, but not T cells, through interaction with RUNX3 and formation of chromatin loops. OCR369 deletion resulted in a significant reduction of RORγt^+^ APCs in mLN around the weaning period and ILC3s in mLN and intestines of adult mice, accompanied by decreased RORγt^+^ Tregs and spontaneous inflammation in the small intestine. Mechanistically, the reduction in RORγt^+^ APCs, including both DC-like cells and MHCII^+^ ILC3s, impaired the development of both dietary antigen-specific and microbiota-specific RORγt^+^ Tregs and resulted in a loss of oral tolerance, thereby increasing allergy susceptibility. Thus, our findings identify a specific regulatory mechanism for RORγt expression in RORγt^+^ APCs and underscore the pivotal role of these cell types in mediating oral tolerance and maintaining intestinal health.

## Introduction

Group 3 innate lymphoid cell (ILC3s), which are enriched in the gut and gut-associated lymphoid tissues, play a critical role in protecting the host from various infections and maintaining intestinal homeostasis^1–5^. ILC3s can directly or indirectly regulate various cells within the intestinal microenvironment^6–8^. Notably, ILC3s express major histocompatibility complex class II (MHCII) and are capable of antigen processing and presentation^9^. Unlike traditional antigen-presenting cells (APCs) such as dendritic cells (DCs), intestinal MHCII^+^ ILC3s lack the expression of co-stimulatory molecules like CD80 and CD86 at steady state, which tends to prevent the over-activation of CD4 T cells, thereby contribute to the maintenance of immune homeostasis in the intestine, tumors, and during allergic airway inflamamtion^9–12^. However, under inflammatory condition, ILC3 subset can upregulate these molecules and become pro-inflammatory^13^. ILC3s also promote the differentiation and survival of commensal-specific RORγt^+^ Treg cells through multiple mechanisms, including OX40L-OX40 interaction, integrin-mediated TGFβ release, and the production of IL-2 and CTLA-4 by ILC3s^14–17^. Recent studies have expanded the group of RORγt^+^ MHCII^+^ APCs, identifying new groups of Thetis cells (TC I-IV) or Janus cells, which are characterized as AIRE^+^ CCR6^+^, sharing some overlapping profiles with ILC3s and also may contribute to the generation of RORγt^+^ Treg cells^18–20^. However, further research is needed to better understand the unique characteristics of these innate RORγt^+^ APCs and to develop methods for their precise identification. Additionally, intestinal Tregs mediate oral tolerance and preventing food allergy, but it is unknown whether RORγt^+^ APCs regulate dietary antigen-specific Tregs and promote oral tolerance.

ILC3s are innate-adaptive counterparts to adaptive Th17 cells, sharing similar lineage-gene profiles and functional roles. RORγt, a master lineage transcription factor (TF), regulates the development of both ILC3s and Th17 cells, and is also required for the survival of CD4^+^CD8^+^ double positive (DP) thymocytes and TCRα rearrangement^21^. Despite of the similar requirement of RORγt, emerging evidence highlights distinct regulatory mechanism between ILC3s and T cells^22,23^. Th17 cell differentiation is driven by RORγt, which relies on STAT3 signaling in response to IL-6 and IL-23 stimulation^24,25^. In contrast, the mechanism underlying RORγt expression initiation in ILC3s from their progenitors remains poorly understood. The development of ILC3s appears to be independent of STAT3^23^, and even transient inhibition or ablation of RORγt has limited effects on the function and population of mature ILC3s^26^, indicating distinct requirements and regulatory mechanisms of RORγt. Recent studies identified several cis-regulatory elements (CREs) at the *Rorc* locus, like conserved non-coding sequence (CNS) 6, CNS9, CNS11 and RORCE2, which play important roles in regulating RORγt expression in T cells^27–29^. However, whether ILC3-specific CREs exist to regulate RORγt expression remains largely unknown.

In this study, we identified an open chromatin region (OCR369) as a specific CRE that regulates high-level RORγt expression in ILC3s and related RORγt^+^ APC subsets. Importantly, OCR369 intrinsically controls RORγt expression in ILC3s but not Th17 cells. Furthermore, OCR369 deletion impaired the development and function of ILC3s and other RORγt^+^ APC subsets, leading to disrupted Treg-induced oral tolerance and increased susceptibility to food allergy. These findings reveal that a distinct CRE can specifically regulate RORγt^+^ APCs, including ILC3s, to maintain oral tolerance by inducing dietary antigen-specific Tregs.

## Results

### A distinct Open Chromatin Region in the Rorc locus is essential for ILC3s.

**Figure 1:**
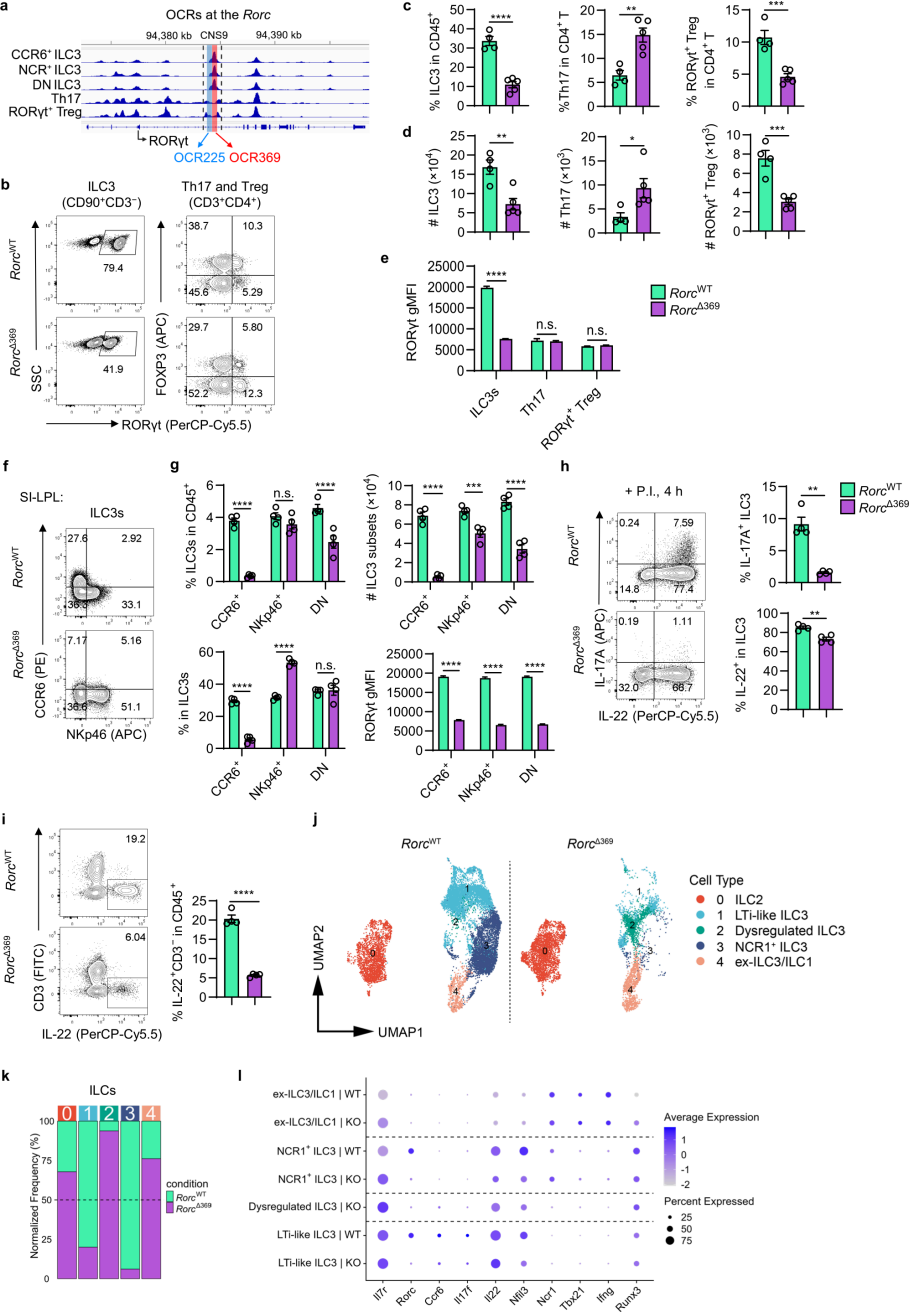
OCR369 is required for high levels of RORγt and ILC3 development. **a**, Comparison of chromatin accessibility (ATAC-seq) at *Rorc* locus among intestinal ILC3s (CCR6^+^ NCR^+^, and DN ILC3s; GSE137319), Th17 cells (GSM3638387), and RORγt^+^ Tregs. OCR225 and OCR369 were identified and named based on their length. **b-e**, Flow cytometry analysis of ILC3s (gated as live CD45^+^CD90^+^CD3^−^RORγt^+^), Th17 cells (live CD45^+^CD4^+^RORγt^+^FOXP3^−^), and RORγt^+^ Treg cells (live CD45^+^CD4^+^RORγt^+^FOXP3^+^) in the small intestine lamina propria of adult mice (*Rorc*^WT^ n=4, *Rorc*^Δ369^ n=5). **c** and **d**, The proportion of ILC3 in CD45^+^ cells, proportion of Th17 and RORγt^+^ Treg in CD4^+^ T cells, and absolute numbers of ILC3, Th17 and RORγt^+^ Treg cells. **e**, The geometric mean fluorescence intensity (gMFI) of RORγt was measured. **f** and **g**, ILC3s in small intestine were further analyzed as NKp46^+^, CCR6^+^, and NKp46^−^CCR6^−^ (DN) subsets, n=4. **h**, The percentage of IL-22^+^ and IL-17A^+^ ILC3s (gated as live CD45^+^CD90^+^CD3^−^RORγt^+^) in total ILC3s from SI, stimulated by IL-23 and IL-1β *ex vivo* for 4 h, n=4. **i**, The percentage of IL-22^+^CD3^−^ cells in live CD45^+^ cells, n=4. **j**, scRNA-seq UMAP visualization of sorted ILCs (gated in live CD45^+^ lineage^−^ (B220, Ter-119, CD11b, Gr-1) CD90^+^ cells) isolated from SI LPL of 6-week-old mice. **k**, Frequencies of ILC cluster 0–4 (shown in Fig. 1j), normalized to total sequenced cells of *Rorc*^WT^ or *Rorc*^Δ369^ mice. **l**, Dot plot showing the average expression and percentage of indicated ex-ILC3/ILC1 and ILC3 clusters. Dysregulated ILC3 cluster was predominantly observed in *Rorc*^Δ369^ mice (KO). Data are representative of three independent experiments (**b-i**), and each symbol represents one mouse (**c**, **d**, **g-i**). Data were analyzed by two-tailed unpaired Student’s t-test and represent Mean ± SEM (**c-e**, **g-i**), n.s., no significance, *p < 0.05, **p < 0.01, ***p < 0.001, ****p < 0.0001.

Chromatin accessibility is closely linked to gene activity, and the landscape of Open chromatin regions (OCR) reflects the regulatory networks specific to distinct cell types. To identify the specific cis-regulatory elements in ILC3s and T cells, we performed ATAC-seq analysis at the *Rorc* locus with intestinal ILC3s, Th17 cells and RORγt^+^ Tregs^22,30,31^ (Th17 from GSM3638386 and ILC3 subsets from GSE137319 of GEO database). While all ILC3 subsets, Th17 cells and RORγt^+^ Tregs showed high chromatin accessibility at the RORγt promoter, two regions (OCR225 and OCR369) with higher accessibility were identified in ILC3s, located downstream of the RORγt Promoter within the first intron (Fig. 1a). Interestingly, these two regions located in the CNS9 (Fig. 1a) identified previously^27^. As a part of CNS, OCR369 also showed higher accessibility in human ILC3s than Th17 cells (Extended Data Fig. 1a), suggesting its potential role as an ILC3-specific CRE regulating RORγt expression in both human and mouse.

To investigate the role of OCR225 and OCR369 in RORγt^+^ cells, we used the CRISPR-Cas9 system to generate germline-depleted mice lacking OCR225 (*Rorc*^Δ225^) and OCR369 (*Rorc*^Δ369^) (Extended Data Fig. 1b). Although no significant differences were observed in the proportion, absolute numbers, or RORγt expression levels of intestinal ILC3s and RORγt^+^ T cells between *Rorc*^Δ225^ and wild type mice (Extended Data Fig. 1c, d), *Rorc*^Δ369^ mice exhibited reduced RORγt^+^ ILC3s, increased RORγt^+^ Th17 cells, and decreased RORγt^+^ Tregs in the small intestine (Fig. 1b–d). This contrasts with findings in CNS9-deficient mice^27^, indicating a distinct regulatory mechanism. Wild-type ILC3s showed much higher RORγt level than Th17 cells and RORγt^+^ Tregs, and OCR369 depletion in ILC3s significantly reduced RORγt expression to levels comparable to those in Th17 cells, with no effect on RORγt expression in Th17 or RORγt^+^ Tregs (Fig. 1e). In contrast to unchanged ILC1 and ILC2 cell numbers (Extended Data Fig. 1e), *Rorc*^Δ369^ mice displayed reduced RORγt expression and cell numbers across all ILC3 subsets (CCR6^+^, NKp46^+^, and CCR6^−^NKp46^−^ double-negative (DN) ILC3s) in the small intestine, with the most pronounced reduction in CCR6^+^ ILC3s (Fig. 1f, g). Similarly, c-Kit^+^ ILC3s and NRP1^+^ ILC3s were significantly reduced in *Rorc*^Δ369^ mice (Extended Data Fig. 1f), suggesting that OCR369 is critical for maintaining ILC3 numbers, rather than merely influencing surface marker expression. Decreased ILC3 numbers and RORγt expression were also observed in the large intestine and mesenteric lymph nodes (mLN) (Extended Data Fig. 1g-i), indicating OCR369’s broad role in regulating ILC3 population. Additionally, OCR369-deficient ILC3s exhibited reduced production of IL-22 and IL-17, particularly IL-17A (Fig. 1h). While transient RORγt deletion in ILC3s has been reported to preserve CD3^−^IL-22^+^ cells^26^, *Rorc*^Δ369^ mice showed decreased IL-22^+^ ILCs in the small intestine (Fig. 1i), possibly due to reduced total ILC3 numbers. Notably, RORγt expression in thymic DP thymocytes and the development of CD4^+^ and CD8^+^ T cells were unaffected in *Rorc*^Δ369^ mice (Extended Data Fig. 1j), indicating that OCR369 is not necessary for T cell development.

To further investigate the role of OCR369 in RORγt^+^ ILCs and T cells, we performed single-cell RNA sequencing (scRNA-seq) on innate lymphocytes and CD4^+^ T cells sorted from the small intestinal lamina propria of 6-week-old control and *Rorc*^Δ369^ mice (Supplementary Table 1). After quality control, five distinct ILC clusters and five T cell clusters were identified based on marker gene expression (Extended Data Fig. 2a) and visualized using uniform manifold approximation and projection (UMAP) analysis (Fig. 1j and Extended Data Fig. 2b). *Rorc*^Δ369^ mice exhibited a significant reduction in the normalized frequencies of ILC3 clusters, including both LTi-like and NCR1^+^ ILC3s by relative frequencies analysis of these clusters (Fig. 1k). Reduced expression levels of *Rorc*, *Il17f*, and *Nfil3* were also observed in OCR369-deficient ILC3s (Fig. 1l). Interestingly, alongside an increased ex-ILC3/ILC1 cluster, a dysregulated ILC3 cluster emerged in *Rorc*^Δ369^ mice, characterized by downregulation of all ILC3 marker genes, including *Rorc*, *Ncr1*, *Ccr6*, *Il17f*, *Il22*, and *Nfil3* (Fig. 1k, l, and Extended Data Fig. 2a). Moreover, *Rorc*^Δ369^ mice exhibited increased RORγt^+^ Th17 cell cluster and reduced RORγt^+^ Treg cluster by scRNA-seq (Extended Data Fig. 2c). These data suggest that OCR369 is required for ILC3 homeostasis and may also influence T cell homeostasis in the intestine.

### OCR369 is required for RORγt^+^ APCs.

**Figure 2:**
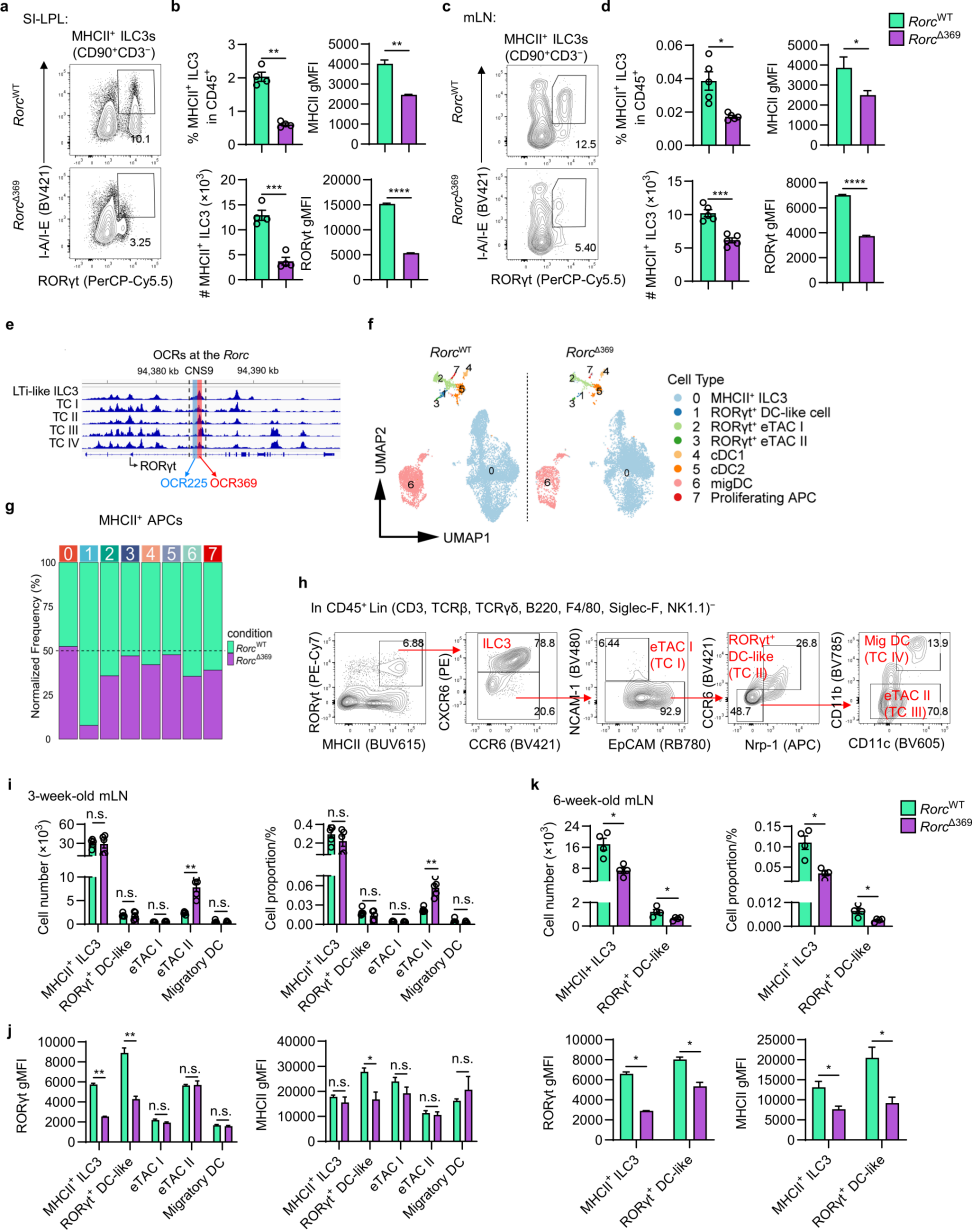
OCR369 regulates multiple RORγt^+^ APC subsets. **a-d**, Proportion, cell number, gMFI of RORγt and MHCII of MHCII^+^ ILC3s (gated in live CD45^+^CD90^+^CD3^−^RORγt^+^MHCII^+^) in small intestine LPL (**a** and **b**, n=4), and mLN (**c** and **d**, n=4). **e**, Comparison of chromatin accessibility (scATAC-seq data) at *Rorc* locus in the LTi-like ILC3 and TC I-IV (GSE174405). **f**, scRNA-seq UMAP visualization of sorted MHCII^+^ APCs (gated in live CD45^+^lineage (Ter-119, B220, NK1.1, Gr-1, F4/80, Siglec-F, TCRβ, TCRγδ, CD3, CD8a)^−^MHCII^+^ cells) isolated from mLN of 18-day-old mice. **g**, Frequencies of ILC cluster 0–7 (shown in Fig. 2f), normalized to total sequenced cells of *Rorc*^WT^ or *Rorc*^Δ369^ mice. **h**, Gating strategy of RORγt^+^ APCs analysis from mLN of 3-week-old mice, gated in live CD45^+^lineage (Ter-119, B220, NK1.1, Gr-1, F4/80, Siglec-F, TCRβ, TCRγδ, CD3, CD8a)^−^. **i**, Cell number and cell proportion in CD45^+^ immune cells of different RORγt^+^ APCs in 3-week-old mice, n=5. **j**, gMFI of RORγt and MHCII in different RORγt^+^ APCs in 3-week-old mice, n=5. **k**, Cell number, cell proportion in CD45^+^ immune cells, gMFI of RORγt and MHCII of different RORγt^+^ APCs from mLN of 6-week-old mice, n=4. Data are representative of three independent experiments (**a-d**, **h-k**), and each symbol represents one mouse (**b**, **d**, **i**, **j**, **k**). Data were analyzed by two-tailed unpaired Student’s t-test and represent Mean ± SEM (**b**, **d**, **i**, **j**, **k**), n.s., no significance, *p < 0.05, **p < 0.01, ***p < 0.001, ****p < 0.0001.

Recent studies have highlighted the importance of MHCII^+^ RORγt^+^ APCs, including MHCII^+^ ILC3s^16^, Janus cells^18^, and Thetis cells^19^, in the development of microbiota-specific RORγt^+^ Treg cells, which are essential for maintaining colon homeostasis^32^. We noticed that MHCII^+^ ILC3s, along with their RORγt and MHCII expression levels, were significantly reduced in the small intestine and mLN of adult *Rorc*^Δ369^ mice (Fig. 2a–d). ATAC-seq analysis of published datasets also showed high chromatin accessibility at OCR369 locus in described Thetis cells (TC I-IV)^18^ (Fig. 2e), indicating OCR369 regulates RORγt expression in RORγt^+^ APCs. To examine the role of OCR369 in RORγt^+^ APCs, lineage-negative (CD3, B220, F4/80, TCRβ, TCRγδ, Siglec-F, Gr-1, Ter-119, F4/80) MHCII^+^ cells were sorted from mLNs of control and *Rorc*^Δ369^ mice at postnatal day 18 (P18) and then subjected to scRNA-seq analysis (Supplementary Table 2). After quality control, the scRNA-seq data was integrated analyzed with published datasets^18,33^ (Extended Data Fig. 3a). Based on marker gene expression and recent annotations, four clusters of RORγt^+^ APCs, including MHC-I^+^ ILC3s, RORγt^+^ DC-like cells (TC II), RORγt^+^ eTAC I (TC I), and RORγt^+^ eTAC II (TC III), were identified (Fig. 2f and Extended Data Fig. 3a, b). MHCII^+^ ILC3s expressed *Rora*, *Rorc*, *Cxcr6*, *Il22*, and RORγt^+^ DC-like cells (TC II) highly expressed *Rorc*, *Prdm16*, *Col17a1*, *Ccr6*, *Nrp1*, while RORγt^+^ eTAC I (TC I) and RORγt^+^ eTAC II (TC III) expressed *Aire* and lower level of *Rorc* (Extended Data Fig. 3b, c). In addition, RORγt^+^ eTAC I highly express *Ncam1* and *Sirpa* (Extended Data Fig. 3b). Although no big difference in the normalized frequencies of MHCII^+^ ILC3s and RORγt^+^ eTAC I/II were observed between control and *Rorc*^Δ369^ group, the normalized frequency of RORγt^+^ DC-like cells was largely reduced in the mLN of P18 *Rorc*^Δ369^ mice (Fig. 2g). Moreover, although the decreased expression of *Rorc* could be detected in both MHCII^+^ ILC3s and RORγt^+^ DC-like cells, only OCR369-deficient RORγt^+^ DC-like cells showed decreased expression of MHCII genes (e.g., *H2-Ab1*, *H2-Aa*, *H2-Dma*, and *H2-DMb1*) (Extended Data Fig. 3c-d), suggesting that OCR369 regulates the antigen-presenting capacity of RORγt^+^ DC-like cells during early life.

To validate the impact of OCR369 on RORγt^+^ APCs, cells from the mLNs of 3-week-old control and *Rorc*^Δ369^ mice were analyzed by flow cytometry (Fig. 2h). While scRNA-seq data suggested reduced RORγt^+^ DC-like cells in *Rorc*^Δ369^ mice, no obvious reduction in the absolute numbers or proportions of all RORγt^+^ APCs was observed (Fig. 2i). However, OCR369 deficiency led to significant reductions in RORγt expression by MHCII^+^ ILC3s and RORγt^+^ DC-like cells, but not by RORγt^+^ eTAC I and eTAC II (Fig. 2j), consistent with scRNA-seq findings. Moreover, reduced MHCII was observed only in RORγt^+^ DC-like cells, not in other RORγt^+^ APCs, including ILC3s (Fig. 2j). Furthermore, RORγt^+^ APCs from 6-week-old control and *Rorc*^Δ369^ mice were also examined by flow cytometry. In 6-week-old *Rorc*^Δ369^ mice, both MHCII^+^ ILC3s and RORγt^+^ DCs exhibited significant reductions in absolute numbers, proportions, and RORγt and MHCII expression levels (Fig. 2k). These results indicate that OCR369 is required for the regulation of both MHCII^+^ ILC3s and RORγt^+^ DC-like cells in adult mice. Notably, MHCII^+^ ILC3s was the most abundant RORγt^+^ APCs in the mLN of both 3-week-old and 6-week-old mice (Fig. 2i, k). Taken together, these data suggest that OCR369 regulates the maintenance and function of RORγt^+^ APC subsets, particularly RORγt^+^ DC-like cells during early life and both MHCII^+^ ILC3s and RORγt^+^ DC-like cells in adults.

### OCR369 intrinsically controls ILC3 development.

**Figure 3:**
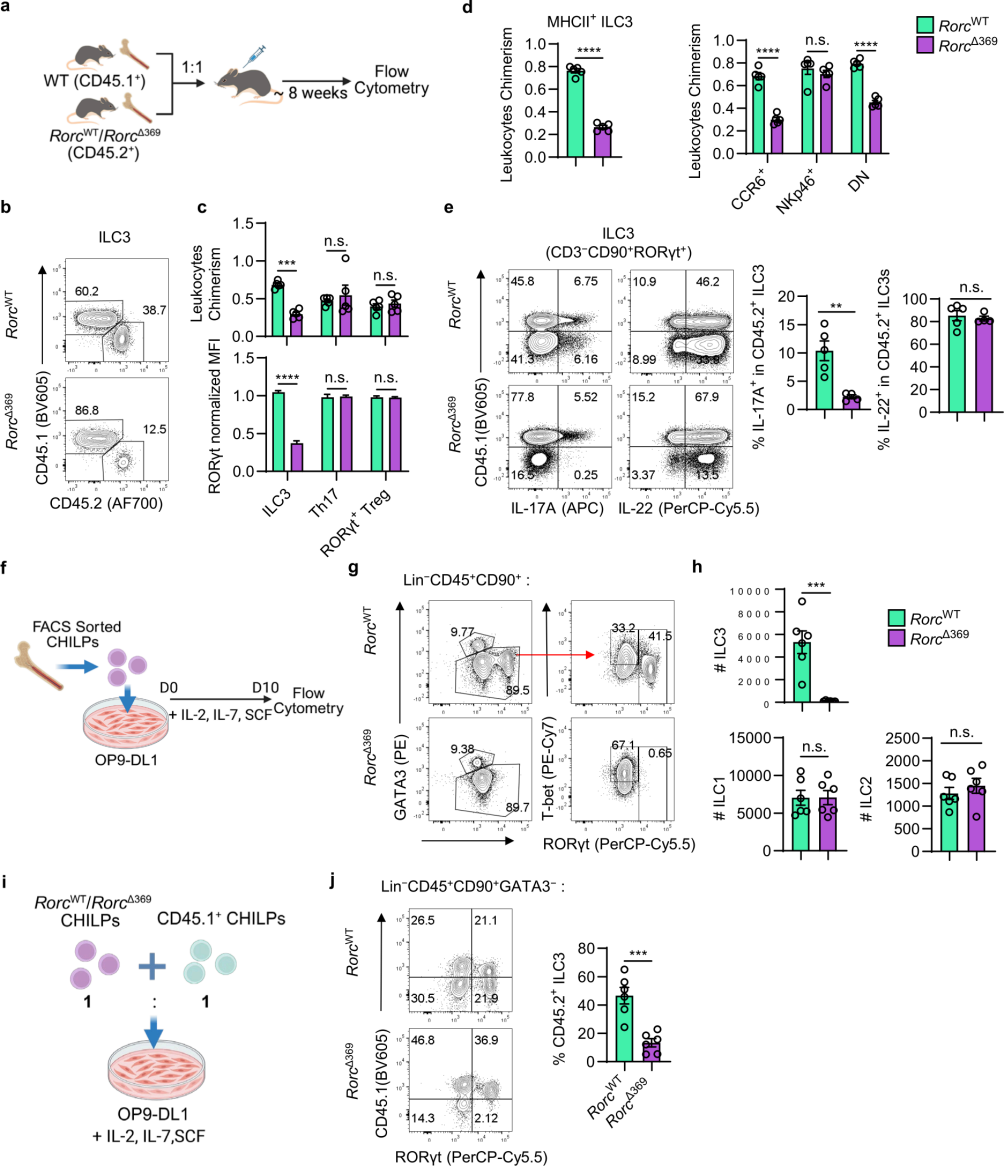
OCR369 intrinsically controls RORγt^+^ ILC3 development. **a**, Schematic design of 1:1 mixed bone marrow chimeric mice: bone marrow cells from *Rorc*^WT^ or *Rorc*^Δ369^ mice were 1:1 mixed with CD45.1^+^ bone marrow cells, then transferred to the irradiated CD45.1^+^ recipients; recipients were sacrificed 8 weeks later for flow cytometry analysis, n=5. **b**, Flow cytometry analysis on intestinal ILC3s (gated in live CD45^+^CD3^−^RORγt^+^). **c**, Statistic results on proportion of ILC3, Th17 and RORγt^+^ Treg cells, normalized by the proportion of CD45.2^+^ splenic B cells (CD45^+^B220^+^) (up). Normalized RORγt gMFI of ILC3, Th17 and RORγt^+^ Treg cells, normalized by RORγt gMFI of CD45.1^+^ control cells (down), n=5. **d**, Normalized proportion of different CD45.2^+^ ILC3 subsets, normalized by the proportion of CD45.2^+^ splenic B cells, n=5. **e**, Percentage of IL-17A^+^ and IL-22^+^ ILC3s in CD45.2^+^ ILC3s (gated in live CD45.2^+^CD90^+^CD3^−^RORγt^+^) after stimulated with IL-23 and IL-1β *ex vivo* for 4 h, *Rorc*^WT^ n=5, *Rorc*^Δ369^ n=4. **f**, Experiment design of sorted CHILP *in vitro* differentiation. Bone marrow CHILPs were sorted (gated in lineage (Ter-119, B220, NK1.1, Gr-1, CD11b, CD3)^−^CD127^+^α4β7^+^CD25^−^Flt3^−^PD-1^−^) from adult littermates, cultured with irradiated OP9-DL1 feeder cells in 96-well plates, added with IL-7, IL-2 and SCF for 10 days, then cells were collected for flow cytometry analysis. **g**, Flow cytometry analysis on ILCs (gated in live CD45^+^lineage^−^ (Ter-119, B220, NK1.1, Gr-1, CD11b, CD3) CD90^+^ cells) derived from CHILPs. **h**, Cell number of ILC1, ILC2, ILC3 derived from CHILPs, n=6. **i**, Experiment design of CD45.2^+^ *Rorc*^WT^ or *Rorc*^Δ369^ CHILPs co-cultured with CD45.1^+^ CHILPs at 1:1 ratio for 10 days to analyze differentiated ILCs. **j**, Proportion of CD45.2^+^ differentiated ILC3 (gated in live CD45.2^+^lineage^−^ (Ter-119, B220, NK1.1, Gr-1, CD11b, CD3) RORγt^+^), n=6. Data are representative of three independent experiments (**a-j**), and each symbol represents one mouse (**a-e**) or one well (**h, j**). Data were analyzed by two-tailed unpaired Student’s t-tests and represent Mean ± SEM (**c-e, h, j**), n.s., no significance, *p < 0.05, **p < 0.01, ***p < 0.001, ****p < 0.0001.

To confirm the intrinsic role of OCR369 in ILC3s, mixed bone marrow chimeric mice were generated on CD45.1 irradiated mice (Fig. 3a). Among CD4^+^ T cells and ILCs, only OCR369-deficient ILC3s and their RORγt expression were significantly decreased, while RORγt^+^ Th17 cells and Tregs remained unaffected (Fig. 3b, c, and Extended Data Fig. 4a). Further analysis of ILC3 subsets revealed heterogeneous effects of OCR369, which decreased CCR6^+^ and DN ILC3s, particularly MHCII^+^ ILC3s, but not NKp46^+^ ILC3, in *Rorc*^Δ369^ chimeric mice (Fig. 3d). This suggests varying RORγt requirements among ILC3 subsets. In the mLN, OCR369-deficient MHCII^+^ ILC3s exhibited reduced proportion and RORγt expression, while other CD127^−^CXCR6^−^RORγt^+^ APCs remained unaffected in *Rorc*^Δ369^ chimeric mice (Extended Data Fig. 4b). Furthermore, IL-17A, but not IL-22, was significantly reduced in OCR369-deficient ILC3s (Fig. 3e), consistent with previous report of limited RORγt effect on IL-22 production on ILC3s^26^. Overall, these results confirm the specific and intrinsic role of OCR369 in ILC3s and their RORγt expression.

In adult mice, ILC3s develop from bone marrow progenitors, and RORγt expression is essential for their differentiation^34^. To determine how OCR369 influences ILC3s, ILC progenitors including CLPs, CHILPs, and ILCPs, were examined, and we found no differences in their percentages or numbers between *Rorc*^Δ369^ and control mice (Extended Data Fig. 4c, d). When CHILPs were sorted and co-cultured with OP9-DL1 feeder cells to generate ILCs (Fig. 3f), OCR369-deficient CHILPs could develop ILC1 and ILC2 normally, but failed to generate ILC3s in contrast to control CHILPs (Fig. 3g, h). *In vitro* co-culture of CD45.2^+^ OCR369-deficient CHILPs with CD45.1^+^ control CHILPs confirmed that OCR369 deficiency significantly impaired ILC3 development (Fig. 3i, j), indicating that high RORγt expression, controlled by OCR369, is essential for *in vitro* ILC3 development. Meanwhile, naïve CD4^+^ T cells were sorted and cultured under Th17 cell- or Treg-polarizing conditions. Both Th17 and Treg cell were normally differentiated after OCR369 deletion (Extended Data Fig. 4e-g). No cell number differences in IL-17^+^ Th17 cells or RORγt^+^ Tregs were observed, and there was a slight reduction in RORγt^+^ Th17 cells (Extended Data Fig. 4e-g). Considering the reduced RORγt^+^ Tregs and increased Th17 cells in *Rorc*^Δ369^ mice (Fig. 1b–d), these data suggest that OCR369 extrinsically regulates Th17 cells and RORγt^+^ Tregs through its effects on RORγt^+^ APCs. Taken together, these results demonstrate that OCR369 intrinsically controls high RORγt expression and development of ILC3s but does not affect CD4^+^ T cells.

### OCR369 interacts with RUNX3 and chromatin loop formation at the Rorc locus.

**Figure 4:**
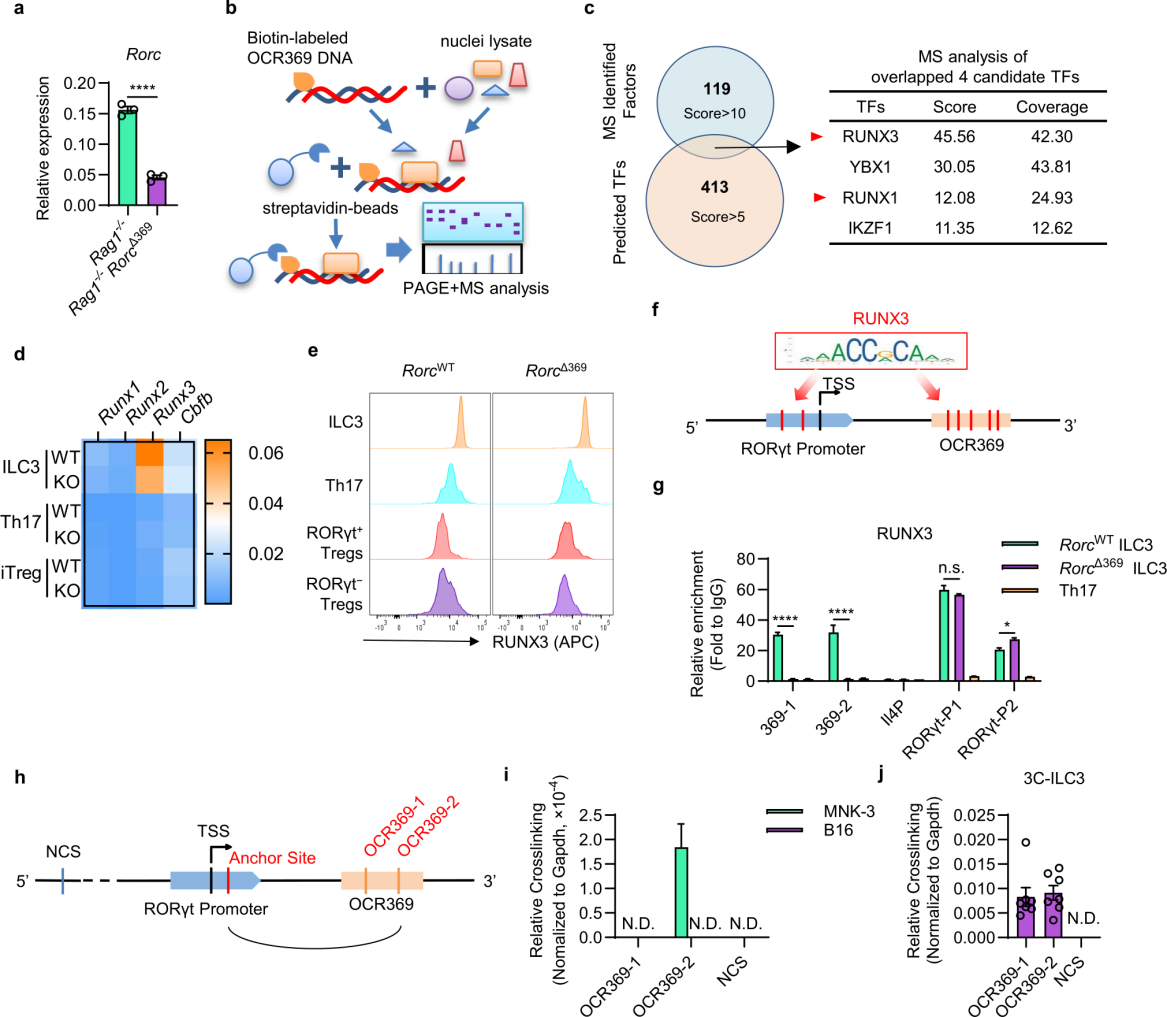
OCR369 interacts with RUNX3 and chromatin loop formation at the *Rorc* loci. **a**, RT-qPCR confirmation of *Rorc* transcripts on sorted ILC3s (lineage (CD3, CD11b, B220, Ter-119, Gr-1, NK1.1) ^−^KLRG1^−^CD90^hi^CD45^lo^) from adult *Rag1*^−/−^ *Rorc*^Δ369^ mice or *Rag1*^−/−^ control mice, n=3. **b**, Schematic design of DNA-pull down assay followed with MS identification: PCR-amplified biotin-labeled OCR369 fragments were incubated with nuclei lysate of sorted ILC3s, then pulled down by the streptavidin-beads, followed by PAGE analysis and MS identification. **c**, Common TFs in the overlap between the TFBS prediction on JASPAR Database (bottom) and MS identification (top). **d**, RT-qPCR heatmap of Runx family transcripts in the intestinal ILC3 (sorted from *Rag1*^−/−^ mice (WT) or *Rag1*^−/−^*Rorc*^Δ369^ mice (KO), gated in lineage (CD3, CD11b, B220, Ter-119, Gr-1, NK1.1)^−^KLRG1^−^CD90^hi^CD45^lo^) and *in vitro* differentiated Th17 and iTreg cells from naïve CD4^+^ T cell from *Rorc*^WT^ (WT) and *Rorc*^Δ369^ (KO) mice. **e**, Flow cytometry analysis of RUNX3 level in the intestinal ILC3 (CD45^+^CD3^−^RORγt^+^), Th17 (CD45^+^CD3^+^CD4^+^RORγt^+^) and RORγt^+^ Tregs (CD45^+^CD3^+^CD4^+^RORγt^+^FOXP3^+^) and RORγt^−^ Tregs (CD45^+^CD3^+^CD4^+^RORγt^+^FOXP3^+^). **f**, The binding motif of RUNX3 and the prediction of RUNX3-binding sites (red bar) in the RORγt-promoter (RORγt-P) and OCR369. **g**, CUT&Run analysis of RUNX3 on RORγt-promoter (RORγt-P1, P2) and OCR369 (369–1, 2) in *Rorc*^WT^ and *Rorc*^Δ369^ ILC3 and *in vitro* differentiated Th17 cells. *Il4* promoter (Il4-P) was used as negative control. Data were normalized with Ct value of IgG Isotype control, represented from two independent experiments. **h**, Schematic map of investigated interaction between RORγt-promoter and OCR369 in 3C-qPCR analysis. NlaIII was used for detecting local chromatin interaction, and the interaction between the anchor NlaIII site with other sites at the *Rorc* locus was detected. Two NlaIII sites in the OCR369 (OCR369–1,2) and a negative control NlaIII site (NCS) over 10 kb upstream of *Rorc* were utilized as previously used^29^. **i** and **j**, Representative result of relative crosslinking of the investigated sites to the anchor site in MNK-3 cell line (**i**), negative control B16 cell line (**i**) (n=7, pooled from two independent experiments), and sorted intestinal ILC3s (**j**) (n=7, pooled from two independent experiments). Data are representative of at least two independent experiments (**a**, **d**, **e**, **g**, **i**, **j**), and each symbol represents one mouse (**a**) or technical replicate (**g**, **i**, **j**). Data were analyzed by two-tailed unpaired Student’s t-tests (**a**), two-way ANOVA with multiple comparisons on indicated groups (**g**) and represent Mean ± SEM, N.D., not detected, n.s., no significance, *p < 0.05, **p < 0.01, ***p < 0.001, ****p < 0.0001.

To determine how OCR369 specifically regulates high RORγt expression in ILC3s, the mRNA level of RORγt was firstly detected. RT-qPCR analysis showed significantly decreased RORγt mRNA in OCR369-deficient ILC3s compared to controls (Fig. 4a), indicating that OCR369 regulates RORγt expression at the transcription level. The binding of specific TFs with cis-regulatory elements is one of the key mechanisms for CREs regulating gene expression. To determine TFs binding to OCR369, the biotin-labeled OCR369 DNA or control *Rorc*(γt) promoter DNA was used to pull-down the specific binding proteins from ILC3 nuclei, followed by mass-spectrometry (MS) analysis (Fig. 4b, Extended Data Fig. 5a and Supplementary Table 3). Combining MS results with TF binding site (TFBS) prediction from JASPAR database (Supplementary Table 4), four candidate TFs were identified, two of which belonged to the RUNX family: RUNX3 and RUNX1 (Fig. 4c). RUNX1 and RUNX3 share similar binding motifs, which are conserved between mouse and human. RUNX3 has been previously reported to be the most abundant RUNX family member in ILC3s, playing an essential role during ILC3 development through directly binding the *Rorc* promoter and inducing RORγt expression^35^. We confirmed that *Runx3* expression was significantly higher than that of *Runx1*, *Runx2*, and *Cbfb* in ILC3s, and that RUNX3 expression was higher in ILC3s compared to Th17 cells, iTregs, and other ILCs (Fig. 4d, Extended Data Fig. 5b, c). In the small intestinal lamina propria, ILC3s expressed higher RUNX3 levels than Th17 cells and Tregs (Fig. 4e). Consistent with predicted RUNX3 binding sites at OCR369 and the RORγt promoter (RORγt-P) (Fig. 4f), CUT&RUN assay revealed stronger RUNX3 binding to both OCR369 and the RORγt promoter in ILC3s compared to Th17 cells (Fig. 4g). OCR369 deficiency significantly influenced RUNX3 binding at OCR369 sites but not at the promoter (Fig. 4g). To test the effect of RUNX3 binding to OCR369 on RORγt expression, a Dual-Luciferase Reporter system with RORγt promoter and OCR369 in HEK293T cells was utilized (Extended Data Fig. 5d). As previously reported^35^, RUNX3 significantly enhanced RORγt promoter activity (Extended Data Fig. 5d). However, the addition of OCR369 did not further enhance transcription (Extended Data Fig. 5d), likely due to the limitations of *in vitro* reporter systems for studying distal cis-regulatory elements. Additionally, RUNX3 is expressed by other RORγt^+^ APCs, particularly RORγt^+^ DC-like cells (TC II) (Extended Data Fig. 5e), suggesting its potential role in regulating the RORγt^+^ APC family. Together, these results indicate that OCR369 interacts with RUNX3 specifically in ILC3s, contributing to the higher RORγt expression in ILC3s compared to Th17 cells.

Chromatin looping has been well recognized as a way for enhancer-promoter interaction to promote gene expression. Since OCR369 is in the first intron, ~ 7kb downstream of the *Rorc* transcription start site (TSS), it may form a chromatin loop with the *Rorc* promoter to enhance RORγt expression in ILC3s. To test this hypothesis, a chromosome conformation capture-qPCR (3C-qPCR) assay was performed in MNK3 cells^36^, an ILC3-like cell line with high chromatin accessibility at OCR369 (Extended Data Fig. 5f). Using the restriction enzyme NlaIII, which was previously used to study chromatin interactions at the *Rorc* locus^29^, two NlaIII restriction recognition sites (OCR369–1, 2) within OCR369 and one at the *Rorc* promoter were analyzed as the anchor site (Fig. 4h). Another upstream NlaIII site without interaction with the *Rorc* promoter was set as a negative control site (NCS)^29^. Compared to the negative control, chromatin crosslinking between OCR369 and the *Rorc* promoter was detected in MNK3 cells but not B16 melanoma cells (Fig. 4i), indicating the formation of the chromatin loop between OCR369 and the *Rorc* promoter in MNK3 cells. This interaction was further confirmed in ILC3s isolated from mouse small intestine (Fig. 4j). Together, these data suggest that OCR369 is involved in the formation of chromatin higher-order structures, which contributes to enhanced expression of RORγt in ILC3s.

### OCR369-deficient mice develop spontaneous small bowel inflammation.

**Figure 5:**
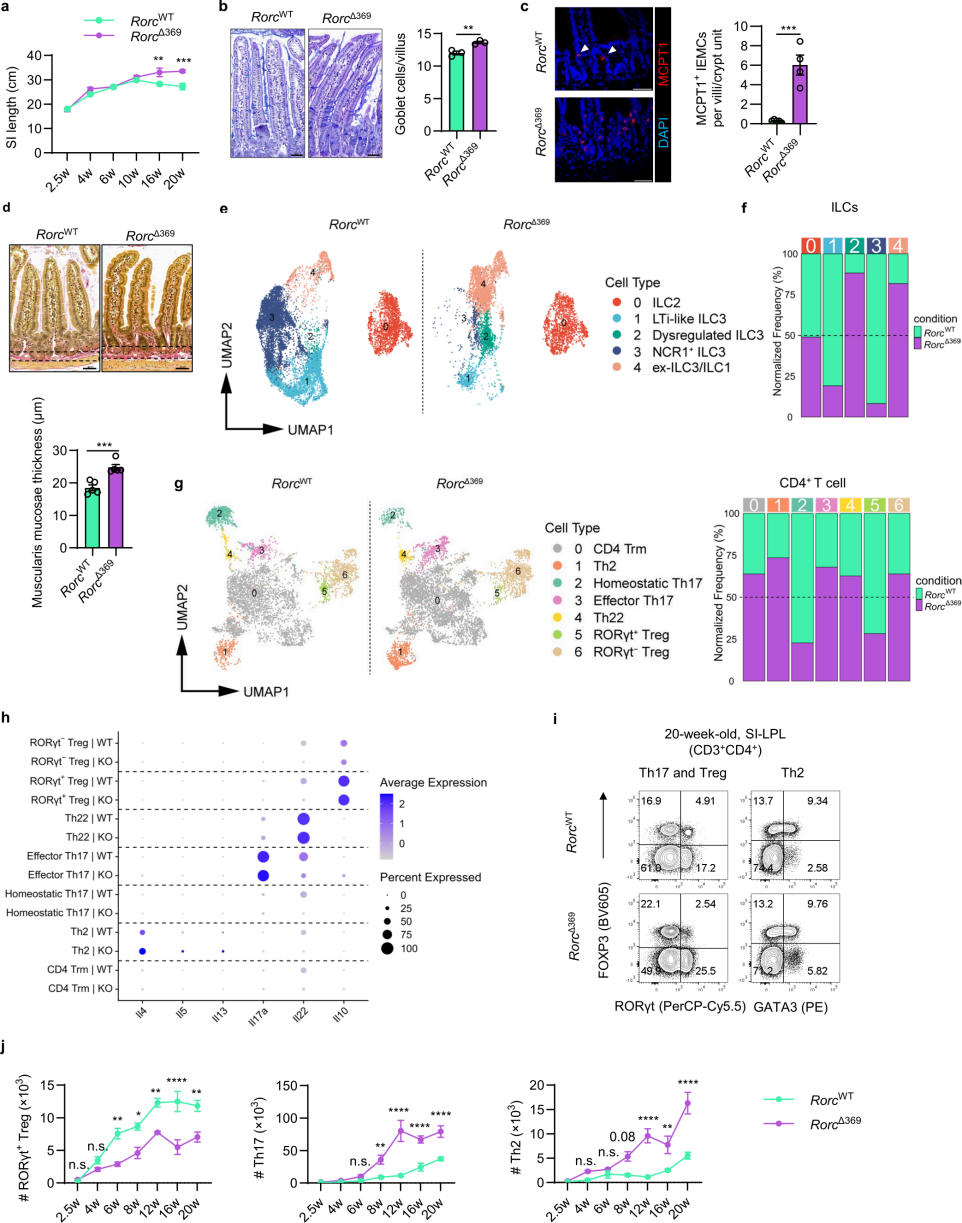
OCR369-deficient mice developed spontaneous inflammation in the small intestine. **a**, Statistic results of the small intestine length of *Rorc*^Δ369^ mice and littermate controls at different ages, n=4 mice at 2.5 w, 4 w, 16 w, n=4–5 at 6 w, n=5 at 10 w, n=3 at 20 w. **b**, Representative image and calculation of goblet cells per villus stained by Alcian Blue-PAS in the small intestine of 20-week-old *Rorc*^Δ369^ mice and littermate controls, n=3, scale bar=50 μm. **c**, Immunofluorescence staining of MCPT1^+^ intraepithelial mast cells (IEMCs) in the small intestine of 20-week-old mice, (*Rorc*^WT^ n=5, *Rorc*^Δ369^ n=4). White arrows point out the IEMCs at the bottom of villi, scale bar=50 μm. **d**, Sirius Red Staining of collagen deposition in the small intestine of 28-week-old mice, submucosal thickness (marked by dashed line) in red was analyzed, scale bar=50 μm, n=5. **e-h**, scRNA-seq UMAP visualization of ILCs (**e**) and CD4^+^ T cells (**g**). CD45^+^ immune cells were isolated from SI of 28-week-old mice. Frequencies of ILC cluster 0–4 (shown in Fig. 2e) (**f**) and frequencies of CD4^+^ T cells cluster 0–6 (shown in Fig. 2g) (**h**) were shown, normalized to total sequenced cells of *Rorc*^WT^ or *Rorc*^Δ369^ mice. Dot plot of effector cytokines expression of CD4^+^ T cells from 28-week-old *Rorc*^WT^ (WT) and *Rorc*^Δ369^ (KO) mice was also shown. **i** and **j**, Representative low cytometry result on 20-week-old mice and (**j**) the kinetic changes of RORγt^+^ Treg (CD3^+^CD4^+^FOXP3^+^RORγt^+^), Th2 (CD3^+^CD4^+^FOXP3^−^GATA3^+^) and Th17 (CD3^+^CD4^+^FOXP3^−^RORγt^+^) cells of distinct ages. *Rorc*^WT^ n=4, *Rorc*^Δ369^ n=3 at 2.5 w; *Rorc*^WT^ n=4, *Rorc*^Δ369^ n=6 at 4 w; n=4 at 6 w and 8 w; *Rorc*^WT^ n=5, *Rorc*^Δ369^ n=3 at 12 w; *Rorc*^WT^ n=6, *Rorc*^Δ369^ n=5 at 16 w; *Rorc*^WT^ n=4, *Rorc*^Δ369^ n=5 at 20 w. Data are representative of three independent experiments (**a-d**, **i**, **j**), and each symbol represents one mouse (**b-d**). Data were analyzed by two-tailed unpaired Student’s t-tests (**b-d**) or two-way ANOVA with multiple comparisons on indicated groups (**a**, **j**) and represent Mean ± SEM, n.s., no significance, *p < 0.05, **p < 0.01, ***p < 0.001, ****p < 0.0001.

ILC3s play an important role in maintaining intestinal homeostasis and anti-infection immunity. To dissect the in vivo role of OCR369 in the intestine, *Rorc*^Δ369^ mice were orally challenged with *Citrobacter rodentium*. However, no significant differences were observed between OCR369-deficient and control mice in terms of body weight change or fecal pathogen load (Extended Data Fig. 6a-c). Although *Rorc*^Δ369^ mice exhibited defects in total ILC3s, IL-17-producing, and IL-22-producing ILC3s in the colon after infection, IL-17-producing CD4^+^ T cells were significantly increased in *Rorc*^Δ369^ mice (Extended Data Fig. 6d-f), which may compensate for host defense against *C. rodentium* in *Rorc*^Δ369^ mice. Interestingly, compared to their littermate controls, *Rorc*^Δ369^ mice developed spontaneous small intestinal remodeling as they aged, characterized by increased small intestine length (Fig. 5a and Extended Data Fig. 7a), villus length (Extended Data Fig. 7b), and goblet cell and DCLK1^+^ tuft cell numbers (Fig. 5b and Extended Data Fig. 7c). These changes of small intestine resemble type 2 inflammation, such as that seen during helminth infection. Indeed, bulk RNA-seq data from 20-week-old littermates showed increased expression of type 2 immune response genes (e.g., *Pla2g4c*, *Dclk1*, *Mcpt1*) and *Gsdmc* genes in the small intestine of *Rorc*^Δ369^ mice (Extended Data Fig. 7d and Supplementary Table 5), which are also upregulated during helminth infection^37,38^. *Mcpt1*, a marker of the intraepithelial mast cells (IEMCs), was significantly increased in the small intestines of *Rorc*^Δ369^ mice (Fig. 5c), indicating mast cell-driven type 2 inflammation. Gene Ontology (GO) enrichment and Gene Set Enrichment Analysis (GSEA) revealed up-regulation of extracellular matrix (ECM) genes in the small intestine of *Rorc*^Δ369^ mice (Extended Data Fig. 7e-g, and Supplementary Table 6), which was further confirmed by Sirius Red staining showing ECM deposition (Fig. 5d), suggesting progressive fibrosis.

To further understand the intestinal immune changes in *Rorc*^Δ369^ mice, scRNA-seq was performed on small intestine samples from 28-week-old *Rorc*^Δ369^ and control mice (Extended Data Fig. 8a and Supplementary Table 7). Consistent with findings in 6-week-old mice, 28-week-old *Rorc*^Δ369^ mice exhibited a more significant reductions in ILC3 clusters, including both LTi-like and NCR1^+^ ILC3s, along with increased ex-ILC3/ILC1 and dysregulated ILC3 cluster (Fig. 5e, f, and Extended Data Fig. 8b). For CD4 T cells, RORγt^+^ Treg proportion was significantly decreased, while Th2 cell and effector Th17 cell proportions were increased in OCR369-deficient mice (Fig. 5g). Accordingly, the inflammatory effector cytokines, such as *Il4*, *Il5*, *Il13*, *Il17a*, produced by these T cells were increased, while inhibitory cytokine *Il10* was slightly decreased in *Rorc*^Δ369^ mice (Fig. 5h). These data suggest that OCR369 deficiency-induced small intestinal inflammation results from altered T cell balance.

Further analysis of Th cell and ILC3 dynamics at different ages revealed that ILC3 proportions in the small intestine remained relatively stable after weaning, with OCR369 deficiency causing persistent reductions (Extended Data Fig. 9a, b). In control mice, RORγt^+^ Tregs developed post-weaning and increased with age in the small intestine, while Th2 and Th17 cells remained low (Fig. 5i, j, and Extended Data Fig. 9c). However, *Rorc*^Δ369^ mice exhibited a significant reduction of RORγt^+^ Tregs and age-dependent increases in Th2 and Th17 cells (Fig. 5i, j, and Extended Data Fig. 9c). Significantly increased inflammatory cytokine production by T cells and innate cells was also observed in the small intestine of *Rorc*^Δ369^ mice (Extended Data Fig. 9d-f). Given the intrinsic role of OCR369 in supporting the RORγt^+^ APC family but not T cells, these findings indicate that OCR369-dependent RORγt^+^ APCs regulate Th/Treg balance in response to antigens from small intestine, probably dietary or microbiota-derived antigens, thereby maintaining small intestinal homeostasis. Indeed, when *Rorc*^Δ369^ mice were crossed with *Rag1*^−/−^ mice, no difference of the small intestinal or villus length was observed between *Rorc*^Δ369^ *Rag1*^−/−^ and control *Rag1*^−/−^ mice at 20 weeks old (Extended Data Fig. 9g), though *Rorc*^Δ369^ *Rag1*^−/−^ mice still showed reduced ILC3s, particularly CCR6^+^ and DN subsets (Extended Data Fig. 9h, i), suggesting that adaptive immune cells are involved in the small intestinal remodeling in *Rorc*^Δ369^ mice. Collectively, these data indicate that OCR369 deficiency leads to progressive inflammation in the small intestine, likely due to Th2/Th17-mediated immune dysregulation.

### RORγt^+^ APCs instruct T cell responses to dietary and microbiota antigens.

**Figure 6:**
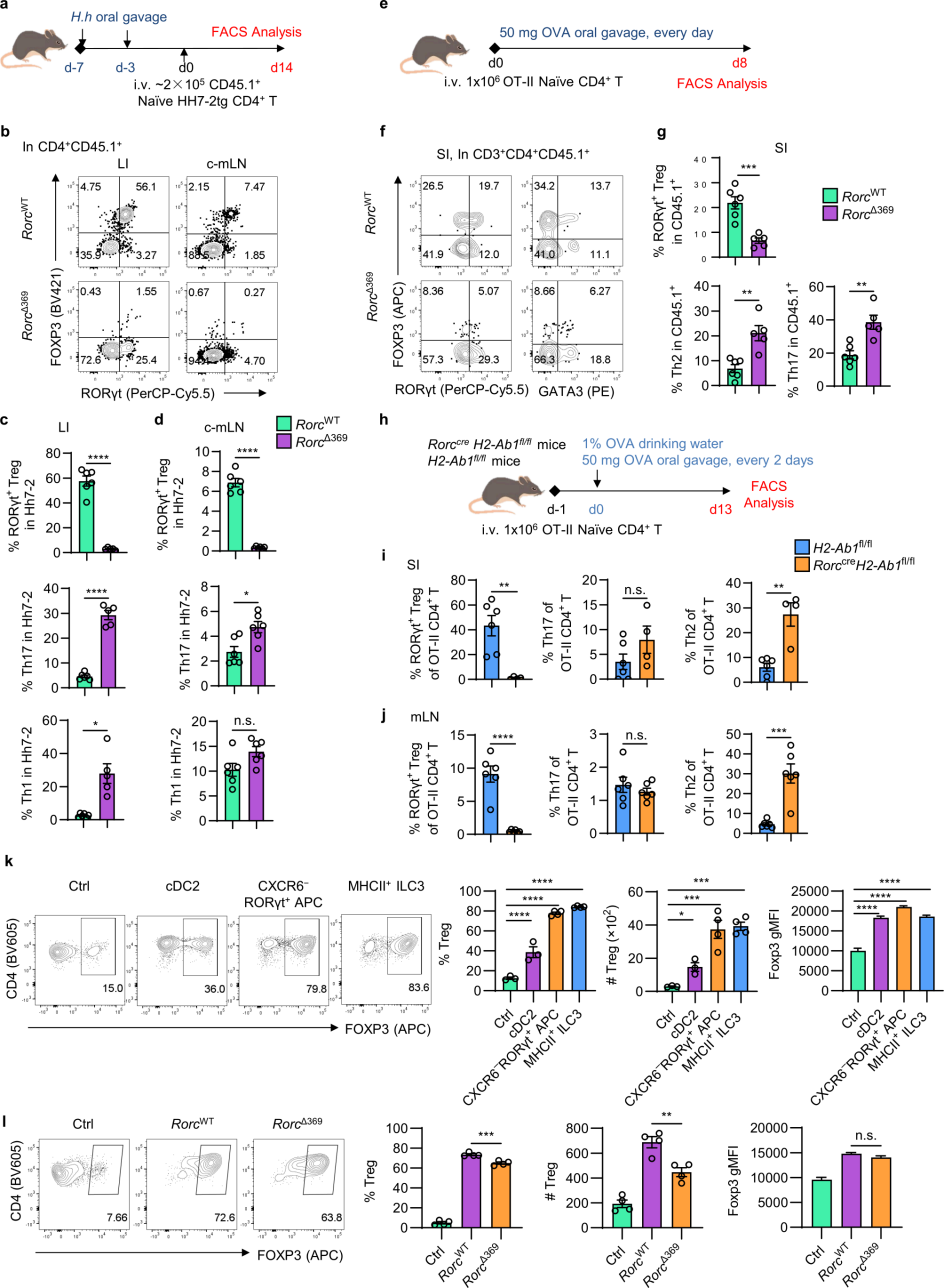
OCR369-dependent RORγt^+^ APC instruct Treg responses to dietary and microbiota-derived antigens. **a**, Schematic design of transferring 2×10^5^ sorted HH-7–2tg naïve CD4^+^ T cell (CD45.1^+^CD3^+^CD4^+^CD44^lo^CD62L^hi^) to the *H. hepaticus* infected (day −7 and day −3) *Rorc*^WT^or *Rorc*^Δ369^ mice. Immune cells were analyzed at day 14. **b-d**, Flow cytometry analysis of the CD4^+^CD45.1^+^Hh7-2tg T cells from large intestine (LI) and colon-draining mLN (c-mLN) (**b**). Proportion of RORγt^+^ Treg (CD3^+^CD4^+^FOXP3^+^RORγt^+^), Th17 (CD3^+^CD4^+^FOXP3^−^RORγt^+^) and Th1 (CD3^+^CD4^+^FOXP3^−^T-bet^+^) in CD4^+^CD45.1^+^Hh7-2tg T cells from LI (**c**, *Rorc*^WT^ n=6, *Rorc*^Δ369^ n=5) and c-mLN (**d**, n=6). **e**, Schematic design of transferring 1×10^6^ sorted OT-II naïve CD4^+^ T cells to the *Rorc*^WT^ or *Rorc*^Δ369^ mice. Mice were orally treated with 50 mg OVA every day and analyzed after 1 week treatment. **f**, Flow cytometry analysis of OVA-specific CD4^+^ T cell response, gated in CD3^+^CD4^+^CD45.1^+^. **g**, Percentage of OVA-specific RORγt^+^ Treg, Th2 and Th17 cell percentage in total CD45.1^+^ OT-II T cells in small intestine, *Rorc*^WT^ n=6, *Rorc*^Δ369^ n=5. **h**, Schematic design for OT-II transferring experiment on *H2-Ab1*^fl/fl^ and *Rorc*^cre^*H2-Ab1*^fl/fl^ mice. **i** and **j**, Proportion of RORγt^+^ Treg (CD3^+^CD4^+^FOXP3^+^RORγt^+^), Th17 (CD3^+^CD4^+^FOXP3^−^RORγt^+^) and Th2 (CD3^+^CD4^+^FOXP3^−^GATA3^+^) in total OT-II T cells in SI (**i**, *H2-Ab1*^fl/fl^ n=6 and *Rorc*^cre^*H2-Ab1*^fl/fl^ n=4) and mLN (**j**, n=6), pooled from two independent experiments. **k**, Naïve CD4^+^ OT-II T cells were cultured alone (Ctrl, n=3), or co-cultured with indicated cDC2 (lineage^–^RORγt-GFP^–^CD11c^+^MHCII^+^, n=3), CXCR6^–^RORγt^+^ APCs (lineage^–^MHCII^+^RORγt-GFP^+^CXCR6^–^, n=4) and CXCR6^+^MHCII^+^ ILC3 (lineage^–^MHCII^+^RORγt-GFP^+^CXCR6^+^, n=4) for 4 days. The percentage of Foxp3^+^ Treg cells amongst total CD4^+^ T cells, cell number and gMFI of Foxp3 in Foxp3^+^ Treg cells were shown. **l**, Naïve CD4^+^ OT-II T cells were cultured alone (Ctrl, n=4), or co-cultured with indicated *Rorc*^WT^ and *Rorc*^Δ369^ MHCII^+^ ILC3 (CD45^+^lineage^–^CXCR6^+^MHCII^+^) for 4 days. The percentage of Foxp3^+^ Treg cells amongst total CD4^+^ T cells, cell number and gMFI of Foxp3 in Foxp3^+^ Treg cells were shown. Data are representative of two (**h-j**) or three (**a-g**, **k**, **l**) independent experiments, and each symbol represents one mouse (**c**, **d**, **g**, **i**, **j**) or one culture well (**k**, **l**). Data were analyzed by two-tailed unpaired Student’s t-tests (**c**, **d**, **g**, **i**, **j**) or one-way ANOVA with multiple comparisons on indicated groups (**k**, **l**) and represent Mean ± SEM, n.s., no significance, *p < 0.05, **p < 0.01, ***p < 0.001, ****p < 0.0001.

Since RORγt^+^ APCs are crucial for the development of microbiota-specific RORγt^+^ Tregs, and OCR369 deficiency reduces multiple RORγt^+^ APC subsets, next we investigated whether *Rorc*^Δ369^ mice exhibited reduced tolerance to gut microbiota. After orally inoculation with *Helicobacter hepaticus* (*H. h*.), a gut pathobiont, and transfer of naïve *H. h*.-specific CD4^+^ T cells from Hh7-2 TCR transgenic (Hh7-2tg) mice, the differentiation of *H. h*.-specific CD4^+^ T cells in both adult *Rorc*^Δ369^ and control mice was examined by flow cytometry (Fig. 6a). In control mice, Hh7-2 T cells mainly differentiated to RORγt^+^ Tregs in both the colon and colon-draining mLN (Fig. 6b–d). However, in *Rorc*^Δ369^ mice, the development of *H. h*.-specific RORγt^+^ Tregs was almost completely abrogated, and Hh7-2 T cells mainly differentiated to inflammatory Th17 and Th1 cells (Fig. 6c), highlighting that OCR369 is essential for RORγt^+^ APCs to instruct microbiota-specific RORγt^+^ Tregs.

Given that OCR369-deficient mice developed spontaneous small intestinal inflammation with reduced RORγt^+^ Tregs and increased Th2 cells (Fig. 5), we explored whether OCR369-dependent RORγt^+^ APCs, particularly RORγt^+^ DC-like cells and MHCII^+^ ILC3s, are also required for the development of dietary antigen-specific Tregs. CFSE-labeled or CD45.1^+^ naïve ovalbumin (OVA)-specific CD4^+^ T cells from OT-II TCR transgenic mice were transferred into *Rorc*^Δ369^ and control adult mice, followed by daily oral OVA administration (Fig. 6e and Extended Data Fig. 10a). At day 4 post OVA challenge, a significant reduction in CFSE^+^ RORγt^+^ Treg cells and an increase in CFSE^+^ Th17 cells in the mLN and peyer’s patches (PPs) were observed in *Rorc*^Δ369^ mice (Extended Data Fig. 10b-d). By day 8, *Rorc*^Δ369^ mice showed fewer CD45.1^+^RORγt^+^ OT-II Tregs and more CD45.1^+^RORγt^+^ OT-II Th17 and CD45.1^+^GATA3^+^ OT-II Th2 cells (Fig. 6f, g). Similarly, endogenous OVA-specific CD45.2^+^ T cells in *Rorc*^Δ369^ mice exhibited reduced RORγt^+^ Tregs and increased inflammatory RORγt^+^ Th17 and GATA3^+^ Th2 cells (Extended Data Fig. 10e-g). The dependence of RORγt^+^ APCs for dietary antigen-specific Treg development was confirmed in mice with specific MHCII deletion in RORγt^+^ APCs (*Rorc*^cre^*H2-Ab1*^fl/fl^), where OVA-specific RORγt^+^ Tregs were nearly absent, and Th2 cells dominated (Fig. 6h–j). Together, these data demonstrate that OCR369-dependent RORγt^+^ APCs are crucial for the development of dietary antigen-specific RORγt^+^ Tregs. Both MHCII^+^ ILC3s and CXCR6^−^RORγt^+^ APCs (including RORγt^+^ DC-like cells) isolated from the mLN of 3-week-old RORγt-reporter mice were capable of promoting *in vitro* OVA-specific iTreg development (Fig. 6k). However, OCR369-deficient MHCII^+^ ILC3s from adult mLN exhibited reduced ability of promoting Treg differentiation (Fig. 6l), emphasizing the importance of OCR369 in regulating the function of RORγt^+^ APCs.

To confirm the role of Tregs in maintaining intestinal morphology and homeostasis, *Rorc*^Δ369^ mice were adoptively transferred with Tregs every 4 weeks starting at 4 weeks of age (Extended Data Fig. 11a). Treg transfer restored small intestine length, villus length, and reduced goblet cell and tuft cell numbers (Extended Data Fig. 11b-e), while significantly reducing Th2 cells in *Rorc*^Δ369^ mice (Extended Data Fig. 11f). Since OCR369 is required for both dietary- and microbiota-specific Treg development, we investigated whether the immunopathology in *Rorc*^Δ369^ mice arises from intolerance to dietary or microbiota antigens. Control and *Rorc*^Δ369^ mice were treated with antibiotics in the drinking water alone, elementary-diet alone or both since born (Extended Data Fig. 12a-c). Elementary-diet treatment, but not antibiotics alone treatment, eliminated the differences of RORγt^+^ Tregs in the small intestine between control and *Rorc*^Δ369^ mice, indicating that OCR369-dependent RORγt^+^ Tregs in the small intestine are induced by dietary antigens. Moreover, only combined treatment with antibiotics and elementary diet controlled Th2 and Th17 cell increases in *Rorc*^Δ369^ mice (Extended Data Fig. 12a-c), indicating that both dietary and microbiota antigens contribute to the intestinal inflammation in OCR369-deficient mice. Together, considering the dynamic influence of OCR369 on RORγt^+^ APCs (Fig. 2), these results indicate that RORγt^+^ APC subsets critically instruct tolerance to dietary- and microbiota-derived antigens in a temporal manner across the lifespan.

### OCR369-dependent RORγt^+^ APCs restrain food allergy

**Figure 7:**
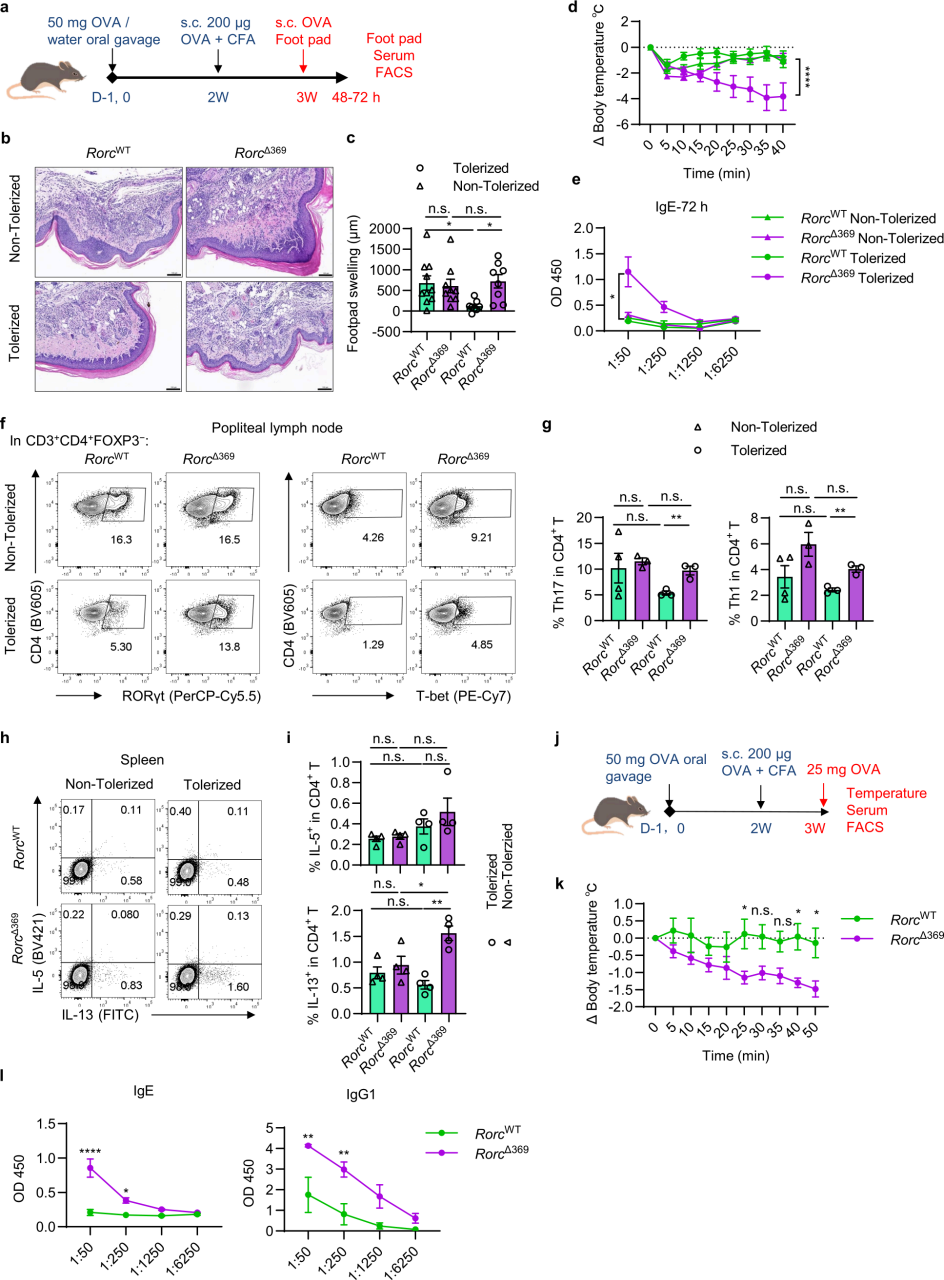
OCR369-deficient mice exhibit impaired oral tolerance. **a**, Schematic design of generating OVA tolerance in the Delayed-type hypersensitivity model (OVA-DTH). 8-week-old mice were treated with 50 mg OVA (Tolerized) or water (Non-Tolerized) by oral gavage twice, and immunized with 200 μg OVA mixed with Complete Freund’s Adjuvant (CFA). After 2 weeks, mice were challenged with OVA in PBS on the left footpad, and the right footpad was injected with PBS as control. **b**, Representative images of H&E staining slices in the footpad after OVA challenge. **c**, Statistic summary of relative footpad thickness at 48 hours after the challenge, pooled from two independent experiments, non-Tolerized *Rorc*^WT^ n=8 and *Rorc*^Δ369^ n=8, Tolerized *Rorc*^WT^ n=10 and *Rorc*^Δ369^ n=9. **d**, Body temperature change of mice after OVA challenge on the footpad, non-Tolerized *Rorc*^WT^ n=5 and *Rorc*^Δ369^ n=5, Tolerized *Rorc*^WT^ n=5 and *Rorc*^Δ369^ n=6) **e**, OVA-specific IgE level in the serum at 72 h after the challenge, x-axis represents the dilution rate of the serum, n=3. **f**, Flow cytometry analysis of Th17 (CD3^+^CD4^+^FOXP3^−^RORγt^+^) and Th1 cells (CD3^+^CD4^+^FOXP3^−^T-bet^+^) in the popliteal lymph nodes (pLNs) of the same side of challenged left footpad, gated in live CD3^+^CD4^+^FOXP3^−^. **g**, The percentage of Th17 (CD3^+^CD4^+^FOXP3^−^RORγt^+^) and Th1 cells (CD3^+^CD4^+^FOXP3^−^T-bet^+^) in CD4^+^ T cells from the popliteal lymph nodes (pLNs) on the same side of challenged left footpad, Non-Tolerized *Rorc*^WT^ n=4 and *Rorc*^Δ369^ n=3, Tolerized *Rorc*^WT^ n=3 and *Rorc*^Δ369^ n=3. **h** and **i**, Percentage of IL-5^+^ or IL-13^+^ CD4^+^ T cells in the splenocytes after ex vivo P.I. stimulation for 4 h, n=4. **j**, The OVA-DTH model was further modified by orally challenging with OVA on the previously “Tolerized” mice. **k**, Body temperature change after the OVA challenge (*Rorc*^WT^ n=5 and *Rorc*^Δ369^ n=6). **l**, Serum OVA-specific IgE and IgG1 level 12 h after the oral challenge (*Rorc*^WT^ n=5 and *Rorc*^Δ369^ n=6). Data are representative of two (**b**, **c**, **h**-**l**) or three (**d**-**g**) independent experiments, and each symbol represents one mouse. Data were analyzed by two-way ANOVA with multiple comparisons on indicated groups (**d**, **e**, **k**, **l**) or two-tailed unpaired Student’s t-tests between indicated groups (**c**, **g**, **i**) and represent Mean ± SEM, n.s., no significance, *p < 0.05, **p < 0.01, ***p < 0.001, ****p < 0.0001.

Given the OCR369-dependence of RORγt^+^ APC and the associated role in regulating Th/Treg balance in response to dietary antigens, we hypothesized that these APCs contribute to oral tolerance beyond microbiota homeostasis. A delayed-type hypersensitivity (DTH) model using OVA (OVA-DTH) was employed (Fig. 7a). OVA feeding before immunization induced tolerance to OVA challenge in control mice, but failed to do so in *Rorc*^Δ369^ mice, as indicated by severe foot pad swelling and immune cell infiltration (Fig. 7b, c). Interestingly, “tolerized” *Rorc*^Δ369^ mice not only displayed elevated Th17 and Th1 cells in the foot pad-draining lymph nodes, but also showed significant systemic allergic responses post challenge, as evidenced by decreased body temperature, increased serum OVA-specific IgE levels, as well as increased Th2 cells and IL-13-producing T cells in the spleen and peripheral blood (Fig. 7d–i, and Extended Data Fig. 13a-f). These findings demonstrate that OVA pre-feeding induces a robust allergic response rather than tolerance in OCR369 deficient mice. Additionally, transferring OT-II T cells into the OVA-DTH mouse model exacerbated the allergic response in *Rorc*^Δ369^ mice, causing rapid body temperature drops and mortality within 30 minutes of OVA challenge (Extended Data Fig. 13g, h). Moreover, *Rorc*^Δ369^ mice exhibited heightened susceptibility to food allergy, with significant body temperature drops, increased serum OVA-specific IgE and IgG1 levels (Fig. 7j–l). Together, these data demonstrate that OCR369-dependent RORγt^+^ APCs are essential for maintaining oral tolerance and preventing food allergy.

## Discussion

Recent advances highlight the critical role of the RORγt^+^ APC family, marked by MHCII expression, in shaping peripheral immunity and tolerance through direct interaction with T cells. The characterization and identification of the cell types is still expanding, and so far the major population includes the MHCII^+^ ILC3s, extra-thymic Aire-expressing cells (eTACs), which resemble the medullary thymic epithelial cells (mTECs) and other potential CXCR6^−^IL-7R^−^ DC-like populations^39^. Recent studies have revealed the essential role of RORγt^+^ APCs in regulating the Th/Treg balance, particularly in generating microbiota-specific RORγt^+^ Treg cells in the intestinal environment^15,16,18,19^. The newly reclassified RORγt^+^ APC family, previously called thetis cells (TC I-IV, probably includes the eTACs and DC-like cells), have revealed a kinetics relationship with the generation of RORγt^+^ Tregs during the weaning stage^18^. Previous findings have painted an inspiring picture of RORγt^+^ APCs being active at the early stage of weaning, when Thetis cells reach their peak abundance before declining to a low proportion in adulthood^18^. Through an integrated analysis of our and previously published scRNA-seq data, we identified four distinct clusters of RORγt^+^ APCs: MHCII^+^ ILC3s, RORγt^+^ DC-like cells (TC II), RORγt^+^ eTAC I (TC I, Janus cell), and RORγt^+^ eTAC II (TC III). Interestingly, the regulatory effects of OCR369 on different RORγt^+^ APCs vary and are age-dependent. Around weaning, OCR369 is essential for maintaining the population and function of RORγt^+^ DC-like cells, but not other RORγt^+^ APCs, in the mLN during the first 2–3 weeks of life. While OCR369 deletion significantly impacts RORγt expression in MHCII^+^ ILC3s around weaning, it does not alter their cell numbers or MHCII expression levels. These findings suggest that OCR369-dependent RORγt^+^ DC-like cells may be the key RORγt^+^ APCs driving intestinal tolerance and preventing inflammation during early life. In adult mice older than 6 weeks, OCR369 deficiency leads to a dramatic reduction in the numbers and function of both RORγt^+^ DC-like cells and MHCII^+^ ILC3s. Both cell types can promote *in vitro* Treg development, but OCR369 deficiency diminishes the capacity of MHCII^+^ ILC3s in driving Treg development. Our data, along with previous studies, consistently show that MHCII^+^ ILC3s constitute the majority of RORγt^+^ APCs in the mLN since weaning^18,19^. The emergence of Treg/Th imbalances around 6 weeks in *Rorc*^Δ369^ mice, coupled with impaired development of dietary/microbiota-specific RORγt^+^ Tregs in adulthood, supports complementary roles for MHCII^+^ ILC3s and RORγt^+^ DC-like cells in maintaining intestinal tolerance in mature animals. However, further evidence is needed to elucidate the roles of different RORγt^+^ APC subsets in mediating oral tolerance in adults. Additionally, whether adoptive transfer of RORγt^+^ APCs could rescue or enhance oral tolerance remains to be tested. Furthermore, the mechanisms by which RORγt regulates the function of RORγt^+^ APCs are not yet fully understood. Our findings suggest that antigen-presenting capacity is modulated by RORγt through MHCII expression regulation, while other pathways, such as IL-2 production^15^, active TGF-β release via integrin^18,19,40^, have been shown to be important for RORγt^+^ APCs in regulating Tregs. Whether these pathways are also involved in Treg-dependent food tolerance regulation by RORγt^+^ APCs requires further investigation. A notable question is why OCR369 selectively regulates different RORγt^+^ APC subsets, such as ILC3s and RORγt^+^ DC-like cells, and T cells. One plausible explanation is that MHCII^+^ ILC3s and RORγt^+^ DC-like cells express higher levels of RORγt relative to other cells. Notably, RORγt^+^ DC-like cells exhibit the highest RORγt expression, even surpassing ILC3s around weaning. This suggests that OCR369 is required for high RORγt expression, which in turn is critical for the maintenance and function of RORγt^+^ DC-like cells and MHCII^+^ ILC3s. These observations indicate that, in addition to lineage-specific determinants, RORγt expression levels play a crucial role in regulating distinct immune cells, a hypothesis that warrants further validation.

Despite their similar transcription profiles and cytokine production, ILC3s differ from their Th17 cell counterparts in their regulatory mechanism. The differences can be traced to their distinct chromatin landscapes at lineage-determining genes. For RORγt regulation, previous studies has identified multiple CREs, including RORCE2, essential for RORγt induction by STAT3 and SOX-5^29^, and CNS6, indispensable for RORγt expression through IL-6-STAT3 and TGF-β signaling^27^. Herein, we identified OCR369 as a specific CRE amplifying high-level of RORγt expression in ILC3s but not T cells, thereby refining our understanding of distinct CREs regulating type 3 immune cells. The opening of unique cis-regulatory element provides docking sites for TFs that regulate transcription, particularly those conserved in non-coding regions. Here, we find that RUNX3, highly expressed in ILC3s, binds OCR369 in ILC3s but not T cells. Although RUNX3 directly induces RORγt expression and is essential for ILC3 development^35^, the functional implications of its direct binding to OCR369 in ILC3s remain to be investigated. Additionally, whether other TFs interact with OCR369 to promote high RORγt expression is an area requiring further exploration. Moreover, the distinct distribution of multiple CREs is linked to chromatin loop formation anchored by linage-determine TF complexes in higher-order chromatin structures^29,41^. Interestingly, RUNX3 has been identified as a core TF in genome-wide chromatin loop formation in CD8^+^ T cells^42^. In this study, we identified a chromatin loop connecting the *Rorc* promoter and OCR369, suggesting that RUNX3 may collaborate with other key TFs to link multiple CREs and regulate RORγt expression. The involvement of distinct CREs likely fine-tunes RORγt expression kinetics across different cell types, such as RORγt initiation (which requires further investigation) and amplification via elements like OCR369. Furthermore, since RUNX3 is also expressed by RORγt^+^ DC-like cells, and OCR369 is essential for their maintenance and function, it is reasonable to hypothesize that RUNX3 may be required for RORγt^+^ DC-like cells and their contribution to intestinal tolerance.

Overall, we have identified OCR369 as a specific CRE regulating RORγt expression in ILC3s and RORγt^+^ DC-like cells and highlighted the importance of OCR369-dependent RORγt^+^ APCs in oral tolerance induction. These findings provide new insights into potential therapeutic strategies for oral tolerance-related diseases.

## Supplementary Files

This is a list of supplementary files associated with this preprint. Click to download.

- 20240427zhaoetalSupplementaryMaterialscleanZJ.docx
- SupplementaryTable1DifferentiallyexpressedgenesofILCandCD4Tsubsetsfromthesmallintestinallaminapropriaof6weekoldcontrolandRorcXX369mice.xlsx
- SupplementaryTable2DifferentiallyexpressedgenesofantigenpresentingcellsfromthemLNofP18controlandRorcXX369micescRNAseq.xlsx
- SupplementaryTable3MassspectrometryidentifiednuclearfactorsthatbindstoOCR369DNA.xlsx
- SupplementaryTable4TFBSpredictionresultsofOCR369onJASPARdatabase.xlsx
- SupplementaryTable5BulkRNAseqresultsofDEGsreadcountson20weekoldRorcXX369andcontrollittermatemice.xls
- SupplementaryTable6GSEAenrichmentresultsontheRNAseqresultsof20weekoldmice.xlsx
- SupplementaryTable7DifferentiallyexpressedgenesofILCandCD4Tsubsetsfromthesmallintestinallaminapropriaof28weekoldcontrolandRorcXX369micescR
- SupplementaryTable8PrimerlistofqPCRPCRandCRISPRinthisarticle.xlsx
